# Cleaner fish recognize self in a mirror via self-face recognition like humans

**DOI:** 10.1073/pnas.2208420120

**Published:** 2023-02-06

**Authors:** Masanori Kohda, Redouan Bshary, Naoki Kubo, Satoshi Awata, Will Sowersby, Kento Kawasaka, Taiga Kobayashi, Shumpei Sogawa

**Affiliations:** ^a^Laboratory of Animal Sociology, Department of Biology and Geosciences, Graduate School of Science, Osaka City University, Osaka 558-8585, Japan; ^b^Laboratory of Animal Sociology, Department of Biology, Graduate School of Science, Osaka Metropolitan University, Osaka 558-8585, Japan; ^c^Institute of Zoology, University of Neuchâtel, Neuchâtel CH-2009, Switzerland

**Keywords:** self-awareness, self-face recognition, mirror self-recognition, mental image, photograph mark-test

## Abstract

Some animals have the remarkable capacity for mirror self-recognition (MSR), yet any implications for self-awareness remain uncertain and controversial. This is largely because explicit tests of the two potential mechanisms underlying MSR are still lacking: mental image of the self and kinesthetic visual matching. Here, we test the hypothesis that MSR ability in cleaner fish, *Labroides dimidiatus*, is associated with a mental image of the self, in particular the self-face, like in humans*.* Mirror-naive fish initially attacked photograph models of both themselves and unfamiliar strangers. In contrast, after all fish had passed the mirror mark test, fish did not attack their own (motionless) images, but still frequently attacked those of unfamiliar individuals. When fish were exposed to composite photographs, the self-face/unfamiliar body were not attacked, but photographs of unfamiliar face/self-body were attacked, demonstrating that cleaner fish with MSR capacity recognize their own facial characteristics in photographs. Additionally, when presented with self-photographs with a mark placed on the throat, unmarked mirror-experienced cleaner fish demonstrated throat-scraping behaviors. When combined, our results provide clear evidence that cleaner fish recognize themselves in photographs and that the likely mechanism for MSR is associated with a mental image of the self-face, not a kinesthetic visual-matching model. Humans are also capable of having a mental image of the self-face, which is considered an example of private self-awareness. We demonstrate that combining mirror test experiments with photographs has enormous potential to further our understanding of the evolution of cognitive processes and private self-awareness across nonhuman animals.

## Significance

Some animals have the capacity for mirror self-recognition, but implications for self-awareness remain controversial. Here, we show that cleaner fish, *Labroides dimidiatus*, likely recognize their own mirror image using a mental image of the self-face comparable to humans*.* Mirror-naïve fish frequently attacked photographs of both themselves and strangers. In contrast, after passing the mirror test, aggression against their own photograph and composite photographs of own face/stranger body declined, but aggression remained toward unfamiliar and composite photographs of stranger face/own body. Our results suggest that cleaner fish with MSR ability can recognize their own mirror image based on a mental image of their own face, rather than by comparing body movements in the mirror. This study demonstrates how animals recognize self-images.

Humans have a mental image of their own face and recognize their mirror reflection via self-face recognition (1). Mental images constitute mental states, which comprise a diverse class including beliefs, emotions, desires and intentions, with the latter linked to a private self-awareness (1–4). Demonstrating that nonhuman animals have private self-awareness would require evidence of connected and interacting mental states. Like humans, some animals are capable of mirror self-recognition (MSR; e.g., refs. 1 and 5–7), implying that they may be aware of the self. However, the mechanisms underlying MSR remain unknown and mental images of the self and private self-awareness in nonhuman animals remain controversial topics.

Mirror self-recognition in nonhuman animals was first observed in chimpanzees (5, 6). Gallup hypothesized that animals with MSR capacity have a mentalistic view, i.e., mental states or private self-awareness (1, 5, 6, 8), yet this remains largely untested (3, 4, 9–13). A potentially simpler alternative explanation for MSR is the kinesthetic visual-matching model, which hypothesizes that animals visually match body movements with mirror reflections (considered a public self-awareness; refs. 3 and 4). Explicit tests of these two potential underlying mechanisms are lacking (3, 4, 9, 10, 14), including whether they are mutually exclusive or interact. The taxa capable of MSR is growing and becoming more diverse, including great apes, Asian elephants, dolphins, horses, magpies, and cleaner fish (e.g., refs. 2, 6, 8, 15–19, but see ref. 20). As MSR is now documented across diverse taxa, it is essential that we move beyond simply describing which species can and cannot pass the mirror test and start investigating the mechanisms underlying MSR and the implications for self-awareness.

Humans readily distinguish between photographs of both familiar (including the self) and unknown people by referring to mental images of faces (1). Photographic images are useful tools for testing cognitive mechanisms (i.e., mental image of self-face) and have been utilized in animal studies (e.g., primates, ref. 21; fish, refs. 22–24). Importantly, recognition of the self in a photograph cannot be achieved via a kinesthetic visual-matching mechanism, as photographs are motionless, and self-recognition must therefore occur by a mental image of the self.

There has recently been increasing evidence demonstrating that fish can discriminate between familiar individuals based on facial characteristics (i.e., true individual recognition) and can even identify individual humans faces (e.g., refs. 22–29). The cleaner fish, *Labroides dimidiatus*, uses visual cues to identify between different individual members within social groups (30) and has demonstrated MSR ability [passing rate of 94% (17/18 fish tested); refs. 18 and 19]. These fish consequently represent an excellent model study system for testing the mechanisms nonhuman animals use to process MSR. Such a finding would have vast implications for our understanding of the evolution of animal cognition and the mental processes underlying self-awareness in nonhuman animals.

## Results and Discussion

### Mirror Mark Test.

**Fig. 1.**
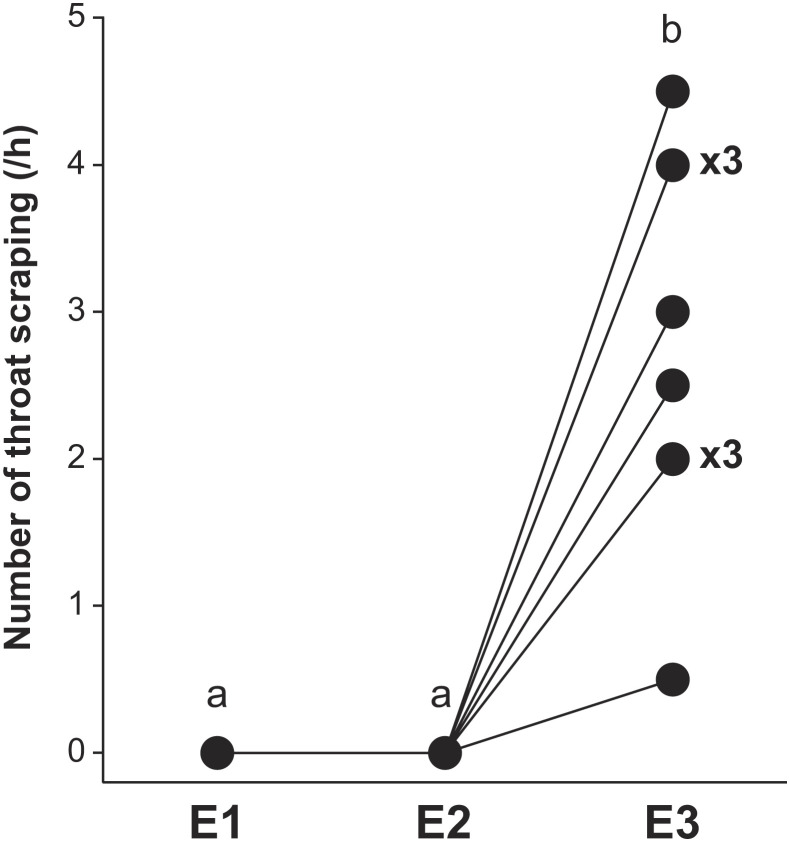
Results of mirror mark test. The number of throat-scraping behaviors by cleaner fish in E1: period before treatment, E2: fish marked and mirror not visible, and E3: fish marked and mirror visible. ×3 = 3 individuals. Friedman test, χ_5_2 = 20.00, *P* < 0.0001, and Kendall W (effect size) = 1.000. *A* and *B* show statistically significant differences by exact Wilcoxon signed-rank tests with sequential Bonferroni adjustment.

We found that all focal fish (*n* = 10) passed the mirror mark test (Fig. 1 as per refs. 18 and 19). That is, all fish displayed throat-scraping behaviors on tank substrate after observing in a mirror reflection a mark resembling an ectoparasite, which had been placed on their throat (2.85 times ± 0.40 SEM/h, E3; similar result to a previous study 3.11 ± 1.26, *n* = 4, exact Wilcoxon rank sum test, *W* = 19.00, *P* = 0.93, and effect size = 0.038; Fig. 1 and ref. 18). In contrast, no throat-scraping behavior was observed prior to the mark being placed on the throat (E1, Fig. 1) or before the mirror was visible to the fish (E2; Fig. 1). Fish were not observed scraping other parts of their body more frequently before or after exposure to a mirror (Table 1) and the frequency of other behaviors including fin erection and touching the mirror did not differ depending on whether the fish was marked or unmarked. Our results strongly suggest that throat scraping is induced by exposure to a mirror and does not reflect a general change in activity patterns. Moreover, our results clearly indicate that cleaner fish recognize themselves in the mirror reflection and attempt to remove the mark observed in the reflection (see ref. 19 for evidence excluding multimodal sensing of the mark).

**Table 1.** Mean ± SEM cleaner fish behavior in three different treatments (*n* = 10 individuals) and results of statistical tests aimed at detecting differences between treatments

	No treatment	Mark treatment	Mark treatment					
Behavior	With mirror	No mirror	With mirror	χ^^2^^	*V*	df	*P*	Effect size
Frequency of throat scraping (times/hr)	0.00 ± 0.00^a^	0.00 ± 0.00^a^	2.85 ± 0.40^b^	20.00	–	2	<0.0001	1.000
Time spent in posture observing the throat (sec/10min)	22.50 ± 2.50	–	70.50 ± 5.70	–	0.00	1	0.002	0.887
Swimming speed(mm/sec)	88.56 ± 7.83^a^	93.24 ± 6.86^a^	103.32 ± 6.11^b^	38.53	–	2	<0.0001	–
Frequency of face and body scraping (times/hr)	3.05 ± 0.44	3.20 ± 0.35	3.15 ± 0.41	0.06	–	2	0.97	0.003
Frequency of fin spreading (times/10min)	5.80 ± 0.68	6.10 ± 0.60	6.50 ± 0.62	2.88	–	2	0.24	0.144
Frequency of touching mirror with mouth (times/10min)	3.80 ± 0.61	–	3.90 ± 0.74	–	21.50	1	0.98	0.049

^*^A linear mixed model was applied to swimming speed of the subject fish (*n* = 5 measurements per treatment per individual); exact Wilcoxon signed-rank tests to time in posture observing the throat in the reflection and frequency of touching mirror with the mouth; and Friedman tests to the other three behavioral factors. Different letters denote statistically significant differences by multiple comparisons using Tukey contrasts (swimming speed) and exact Wilcoxon signed-rank tests with sequential Bonferroni correction (frequency of throat scraping). a and b show statistic differences.

### Experiment 1: Self-Face Recognition.

**Fig. 2.**
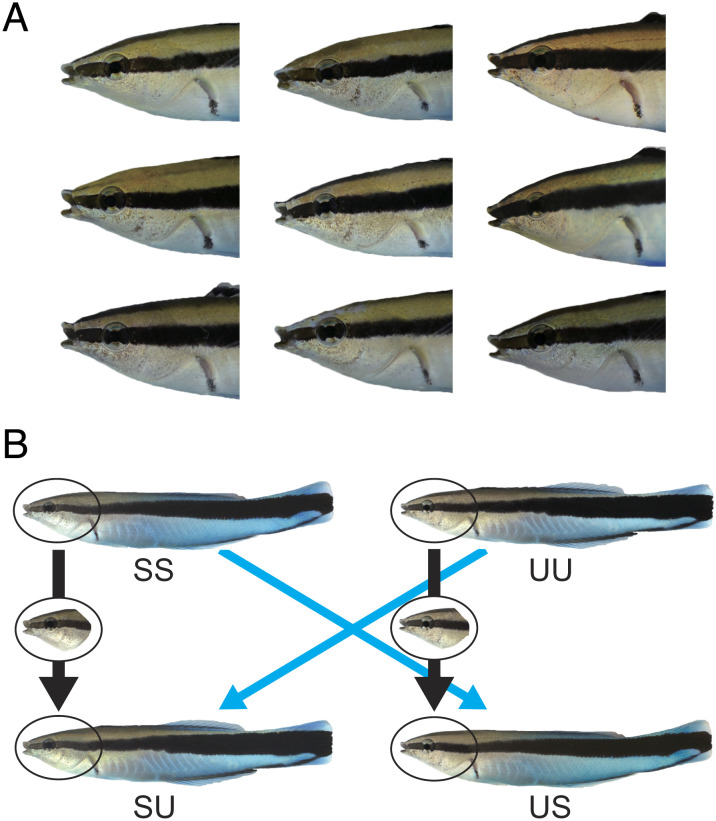
Cleaner fish photograph models used in experiment 1. (Cleaner fish photograph models used in experiment 1. (*A*) Faces of focal fish, and (*B*) an example of the photographic models used in the experiment. SS, self; UU, unknown fish; self-face/unknown body, SU; and unknown face/self-body, US.

**Fig. 3.**
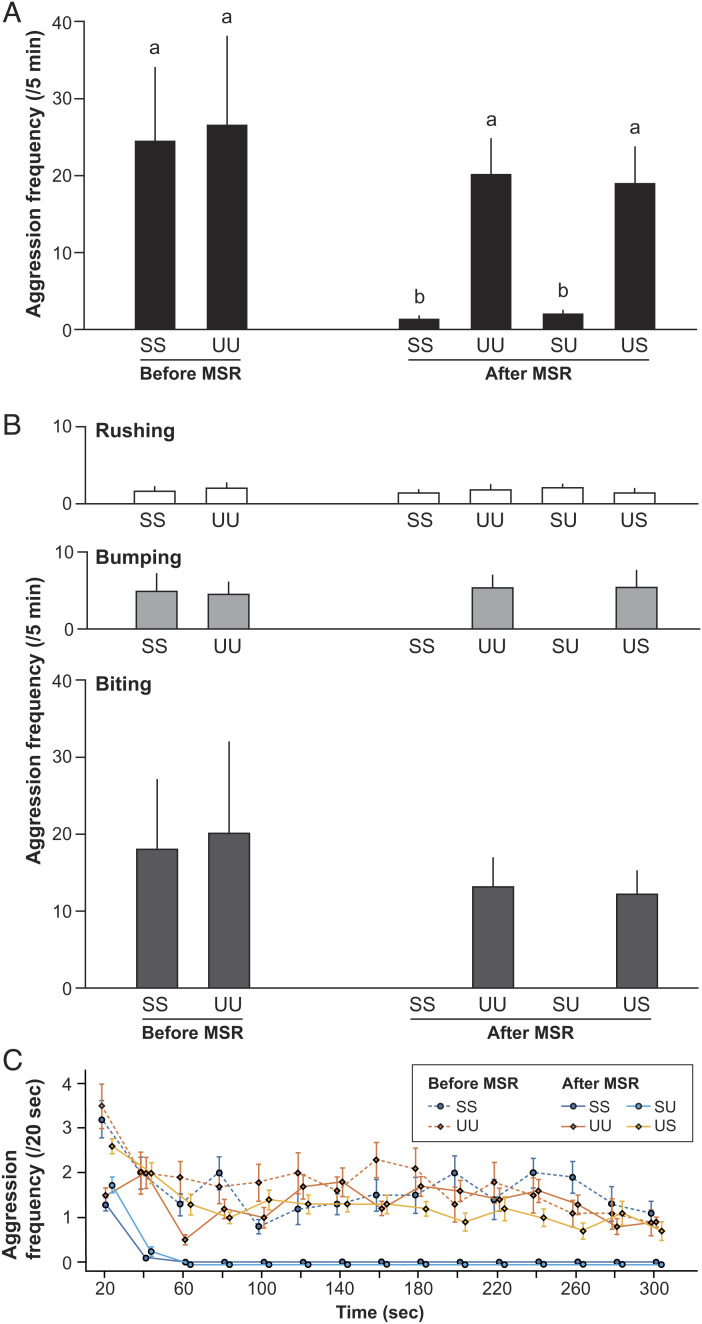
Frequency of aggressive behavior directed toward photographic models in experiment 3. Self (SS) and unfamiliar fish (UU) models and composite self-face/unfamiliar body (SU) and unfamiliar face/self-body (US) models. Before MSR = before mirror presentation, after MSR = after passing the mark test. Means and SEM. (*A*) Total aggression. Friedman test, χ_5_2 = 36.51, *n* = 10, *P* < 0.0001, and Kendall W (effect size) = 0.730. *a* and *b* show statistically significant differences by exact Wilcoxon signed-rank tests with sequential Bonferroni adjustments. (*B*) Aggressive behaviors: rushing (white), bumping (light gray), and biting (dark gray). Friedman test, χ_5_2 = 3.75, *n* = 10, *P* = 0.59, and Kendall W (effect size) = 0.075; χ_5_2 = 27.59, *n* = 10, *P* < 0.0001, and Kendall W (effect size) = 0.552; and χ_5_2 = 35.05, *n* = 10, *P* < 0.0001, and Kendall W (effect size) = 0.701, respectively. (*C*) Changes in aggression toward photograph models over the 5 min experimental period.

Cleaner fish are initially aggressive toward both their own mirror image and unknown individuals (18, 19). As predicted, prior to the mirror mark test, focal cleaner fish also acted aggressively toward their own (Fig. 2) (SS before MSR: 24.5 times/5 min ± 9.5 SEM) and unfamiliar individual photographic images (UU before MSR: 26.6 ± 11.5; Fig. 3*A*). Our results demonstrate that mirror-naive fish do not immediately recognize themselves in a photograph but appear to regard their model images as an unknown territorial intruder.

After passing the mirror mark test, focal fish (*n* = 10) were then randomly exposed to four different composite photographs: self-face/self-body (SS, i.e., own photograph), unfamiliar face/unfamiliar body (UU, i.e., unknown stranger photograph), self-face/unfamiliar body (SU), and unfamiliar face/self-body (US; Fig. 2). Given cleaner fish can visually identify individuals (30), we predicted mirror-experienced focal fish would recognize and act less aggressively toward self-face/self-body photographs, whereas focal fish would regard unfamiliar face/unfamiliar body photographs as unknown strangers. As predicted, all focal fish acted aggressively toward photographs of unknown individuals (UU before MSR: 20.2 ± 4.6; Fig. 3*A*) but not toward their own image (SS before MSR: 1.4 ± 0.3). Importantly, our results suggest that fish can recognize the self-face/self-body photographs as the self and because photographs are motionless, are not doing so via kinesthetic visual matching.

Intriguingly, focal cleaner fish also displayed limited aggression toward composite photographs of the self-face/unfamiliar fish body (SU after MSR: 2.1 ± 0.4) compared with photographs of the unfamiliar face/self-body (US after MSR: 19.0 ± 4.7; Fig. 3*A*). When comparing focal fish responses toward the two different types of self-face photograph models (i.e., self-face/self-body and composite), we observed similar behavioral responses. The frequency of aggression toward the two types of self-face photograph models was significantly lower compared with that of aggression toward the other non–self-face photograph models (i.e., unfamiliar fish model and US), which in turn were not significantly different from each other. Together, these results demonstrate that cleaner fish can recognize themselves in a motionless image and appear to achieve this by self-face recognition.

The aggressive behaviors we observed consisted of three broad types: rushing toward the mirror, bumping with the body, and biting (see *Materials and Methods* for detail). While we did observe that cleaner fish exhibited some aggression toward self-face photographs, this largely consisted of rushing behaviors during the first 20 sec of the 5 min experimental period (Fig. 3 *B* and *C*). In contrast, bumping and biting behavior was directed toward the three types of unfamiliar photographs and self-photographs prior to the mark mirror test throughout the whole 5 min experimental period. We speculate that the motivation behind rushing behaviors toward self-photographs is likely different from the highly aggressive behaviors directed toward unfamiliar photographs and may not represent true aggression (see refs. 18 and 19).

### Experiment 2: Do Cleaner Fish Regard the Self-Face as a Familiar Individual?

**Fig. 4.**
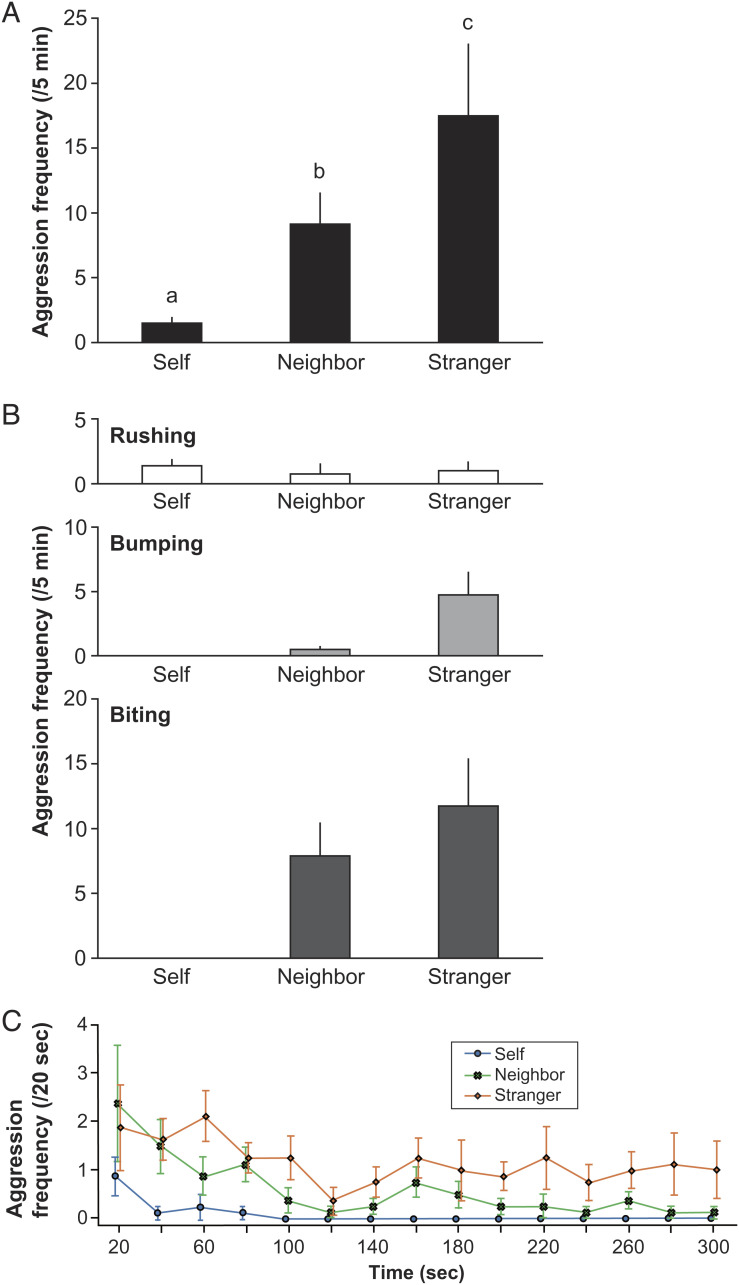
Frequency of aggressive behavior toward three composite photographs of self-face, familiar neighbor face, and unfamiliar stranger face, all placed on the body of another unfamiliar individual. Aggression toward self and neighbor photograph models after a 1 d absence from the focal fish’s territory. Means and SEM. (*A*) Total aggressions. Friedman test, χ_2_2 = 16.00, *n* = 8, *P* < 0.001, and Kendall W (effect size) = 1.000. *A*–*C* show statistically significant differences by exact Wilcoxon signed-rank tests with sequential Bonferroni adjustments. (*B*) Aggressive behaviors: rushing (white), bumping (light gray), and biting (dark gray). Friedman test, χ_5_2 = 3.90, *n* = 8, *P* = 0.14, and Kendall W (effect size) = 0.244; χ_5_2 = 13.04, *n* = 8, *P* < 0.01, and Kendall W (effect size) = 0.815; and χ_5_2 = 14.21, *n* = 8, *P* < 0.001, and Kendall W (effect size) = 0.888, respectively. (*C*) Changes in aggression toward photograph models over the 5 min experimental period.

We next exploited the dear enemy relationship to explicitly test whether focal fish were regarding their self-photograph as a familiar individual, rather than the self. Under the dear enemy relationship, territorial neighbors are tolerated if they are predictable and stay within their own territory (see *Materials and Methods* and refs. 23, 31–33). If a familiar neighbor in an adjoining territory does leave its territory, it is considered to have “betrayed” this relationship, which increases perceived threat levels, leading to increases in aggression (22, 31–33). Focal fish (*n* = 8) with demonstrated MSR ability were exposed to both self-photographs and familiar photographs that we established as potential territorial neighbors that had broken the dear enemy relationship, by leaving an adjoining territory and returning the next day (*sensu* refs. 31 and 32). We predicted that focal fish would recognize self-photographs as the self and not increase aggression when the model returned to the adjoining tank, in comparison with photographs of familiar and unfamiliar individuals (33). Indeed, we found that focal fish aggression did not increase toward the self-photograph (1.5 ± 0.4 SEM) and the frequency of aggressive behaviors was significantly higher toward the photographs of the familiar neighbor (9.1 ± 2.4), which in turn was less frequently attacked than unfamiliar stranger photographs (17.5 ± 5.5; Fig. 4*A*).

Focal fish displayed physical bumping and biting attacks toward both the familiar neighbor and unfamiliar stranger photographs (Fig. 4 *B* and *C*). Aggression declined toward the familiar neighbors over the 5 min experimental period, but remained consistently high toward unfamiliar individuals, suggesting that cleaner fish can also distinguish between familiar and unfamiliar individuals (Fig. 4 *B* and *C* and ref. 30). In contrast, behavioral reactions toward self-photographs typically only comprised rushing behaviors at the start of the experimental period (Fig. 4 *B* and *C*). This is a similar behavioral response to what was observed toward self-photographs in experiment 1, suggesting that no incremental increases in threat level have occurred, as is predicted under the dear enemy relationship if the focal fish regarded the self-photograph as the self. In combination, these results strongly suggest that focal fish regard self-photographs, in particular self-face photographs, as the self. Importantly, we demonstrate that cleaner fish appear capable of having a mental image of the self-face and that they can also recognize the faces of familiar individuals.

### Experiment 3: Photographic Mark Test.

Finally, we presented mark-naive focal fish with self-photographs that had a brown color mark placed on the throat. We speculated that if cleaner fish recognize the self-photograph as the self, marked self-photographs would also elicit throat-scraping behavior, comparable to viewing the marked self in a mirror reflection. Furthermore, throat-scraping behavior elicited by observing a motionless image would strongly suggest that response to a mark on the body is a self-directed behavior and not a response to a perceived familiar individual or driven by kinesthetic visual matching (5, 6, 18, 19). Six of eight mark-naive focal fish displayed throat-scraping behavior after observing a self-photograph with a mark placed on the throat (passing rate 75%; Table 2 and Movie S1). In contrast, no fish scraped their throat when observing a self-photograph that was unmarked or when observing a familiar photograph that was marked. We consider this test a photograph mark test, which in combination with our results from mirror mark tests (refs. 18 and 19, current study) and the results from experiments 1 and 2 provides compelling evidence that cleaner fish recognize self-photographs as the self. Our results also provide clear evidence to suggest that cleaner fish are capable of having a mental image of the self and do not recognize the self via a kinesthetic visual-matching model.

**Table 2.** The number of throat-scraping behaviors (/ h) displayed by cleaner fish in response to a self-photograph with a mark and two control photographs (i.e., self-photograph with no mark and a familiar individual with a mark)

	Self-photograph		Familiar photograph	
Fish ID	With mark	No mark	With mark	Passed test
#1	0	0	0	Unclear
#2	14	0	0	Yes
#3	0	0	0	Unclear
#4	2	0	0	Yes
#5	3	0	0	Yes
#6	1	0	0	Yes
#7	1	0	0	Yes
#8	1	0	0	Yes
Total	22	0	0	

^*^Criteria for passing test: scraping throat only when observing self-photograph with a mark.

## General Discussion

Using a suit of novel experiments, we demonstrate that cleaner fish capable of MSR recognize self-face photographs as the self. Our results provide clear evidence suggesting that these fish can have a mental image of their face after exposure to a mirror and can distinguish their face from the faces of other individuals. In particular, our finding that cleaner fish react to a marked self-photograph demonstrates compelling evidence that cleaner fish achieve self-recognition in images via a mental image of the self rather than a kinesthetic visual-matching process (1, 3, 4, 9, 10, 21). The current study represents the explicit experimental test of the mechanisms underlying MSR in nonhuman animals (4, 5, 34). We do recognize that advanced cognitive abilities and self-awareness in nonprimate animals does remain controversial, particularly in fish, yet our experiments provide robust evidence that like humans, cleaner fish achieve MSR via self-face recognition.

Our results raise an obvious evolutionary question: why would cleaner fish have the capacity to recognize their own face in an image (i.e., in a photograph or mirror reflection)? We know that cleaner fish can recognize the faces of individuals in social groups (30, current study) and we hypothesize that self-face recognition occurs as an extension of this type of individual recognition. The ability to exploit individual-specific differences in faces appears to occur widely across social fishes, to distinguish between familiar and unfamiliar individuals, and to identify specific familiar individuals (22–24, 29, 35, 36). Humans can also rapidly recognize faces in mirrors and photographs by comparing the image against a mental image of the face (1, 25, 37, 38). The ability to quickly and accurately differentiate between individual faces likely has an ancient evolutionary origin in humans and related species (1, 39, 40). We predict that similar selective forces acting on cleaner fish and other social species have led to the evolution of face recognition (30, current study) and consequently the ability to recognize the self-face.

Several animals across multiple taxa have demonstrated MSR, yet the mechanism underlying this ability has until now remained unclear. These species that have passed the mirror mark test are typically social species (e.g., some birds, great apes, and elephants), which often need to visually recognize other individuals to maintain stable social groups (21, 39, 40). We speculate that these species also refer to mental images of individual-specific face characteristics to distinguish familiar from unfamiliar individuals and to identify between certain familiar individuals (25, 38, 39, 40). Considering the potentially similar mental mechanisms used by humans and cleaner fish in MSR processing, we predict that it is probable that other species capable of MSR could also exploit a mental image of the self-face to recognize the self in a mirror image.

According to Gallup 1970 (5), animals with MSR capacity are likely to have a private self-awareness. We demonstrate that cleaner fish appear to refer to a mental image of self-face in order to achieve photograph self-recognition and that they likely obtain the mental image during MSR processing. In social groups, animals need to readily recall previous interactions with other individuals (25, 38) and holding mental images of conspecific faces would facilitate the rapid identification of individuals that have been recently encountered (Fig. 4 and refs. 23, 27, and 38). The ability to recognize faces and then adjust behavior accordingly (e.g., friendly or aggressively) suggests that cleaner fish have the capacity for private self-awareness and mental states associated with mental images of self-face and self-motivation (3, 4).

It is typically assumed that a mental image model and a kinesthetic visual-matching model are alternative mechanistic explanations underlying MSR (3, 4, 9, 10). Our results suggest that developing MSR capacity allows cleaner fish to create a mental image of the self-face, which they can then exploit for visual self-recognition. However, we further speculate that visual matching may provide a crucial initial step toward developing a mental self-image. Specifically, we hypothesize that after the initial aggressive responses to the mirror reflection, cleaner fish then begin to examine the contingency of movement between their body and their mirror reflection, i.e., kinesthetic visual matching (18, 19). This phase of the contingency examination is also documented in other animals that demonstrate MSR, such as chimpanzees, dolphins, and Asian elephants (5, 16, 17, 41). Only after the cleaner fish (and other species with MSR capacity) perceive the reflection as themselves based on contingency testing behaviors, can a mental image of the self-face then develop. What remains untested is how quickly the association between visual matching and a mental image of the self-face forms and whether MSR is possible in this visual-matching phase or whether a mental image of self-face is required.

The original mirror mark test was devised for primates and may therefore yield false-negative results in other taxa (18, 19, 42, 43). To pass the mark test, subject animals are required to complete the following steps: i) notice a mark placed on their body using a mirror reflection, ii) be motivated to touch the mark, and iii) try to physically remove or touch the mark (16–18). Across species, many animals fail the mark test at different steps, for example, because the mark is not perceived as salient or because some species do not have the physical means to touch or try to remove the mark (19, 42, 43). Moving forward, the use of photographs of the self and familiar territorial neighbors (under a dear enemy relationship scenario) and the behavioral responses of focal animals may emerge as alternative techniques for testing self-recognition (Fig. 4), particularly in species unsuited to the traditional mirror mark test (19, 41, 42). Furthermore, the photograph mark test can provide strong evidence to determine whether subjects recognize themselves in self-photographs and in mirrors, which implies a mental image of the self-face.

With the adoption of taxa appropriate methods (18, 19, 42) and the use of self-photographs, we suspect that more animal species will pass self-recognition tests and demonstrate private self-awareness. This approach may resolve several current mismatches between empirical evidence and assumed hierarchies of cognitive complexity. For instance, while it is generally assumed that MSR capacity is a simpler cognitive process than theory of mind (ToM) (3, 4), dogs have so far shown some capacity for the latter but failed in standard MSR tests (3, 4, 44). We believe MSR capability in several species, including dogs, could be observed by employing taxa appropriate methods, potentially including self-photographs.

Gallup claimed that great apes possess MSR ability via a mental image of the self (5, 6, 8, 34). It has, however, also been assumed that language, particularly inner speech, is essential for higher levels of self-processing, including the development of mental states or private self-awareness (e.g., refs. 3, 4, and 45). According to this view, nonlinguistic animals, including the great apes, are incapable of having mental states and hence must process MSR differently to humans (3, 4, 46). Despite over 50 y of research on MSR across various species, the mental mechanisms underlying MSR have not been previously investigated. Potentially, this lack of explicit testing has enforced in the literature the idea that humans have unique mechanisms for MSR processing (3, 4). Yet, by experimentally excluding the kinesthetic visual-matching model, our results suggest that nonlinguistic animals, such as cleaner fish, have the ability for mental states and process MSR comparable to humans. Our results therefore demonstrate the need to reconsider the assumed importance of inner speech for higher cognitive functions, including self-awareness.

There is an increasing number of examples of advanced cognitive capacity in fishes (47, 48). Cleaner fish have emerged as a model system for the study of such advanced cognition, including perspective taking that is related to ToM (49). Nevertheless, there is no compelling reason to suspect that cleaner fish are exceptional within vertebrates or even within fish with respect to the underlying cognitive mechanisms allowing MSR ability (but see ref. 50). Our research suggests that MSR, self-face recognition, and implied mental states may be more abundant among vertebrates than currently appreciated.

## Materials and Methods

### Subject Animals and Housing.

The cleaner fish, *L. dimidiatus*, inhabits coral reefs and rocky areas in the tropical and subtropical Indo-Pacific, where it eats ectoparasites off “client” fish (51). It is a protogynous hermaphrodite, changing sex from female to male, with a harem polygynous mating system (30, 46). Cleaner fish have become a model species for the study of fish cognition (52, 53), yielding evidence for generalized rule learning (54), the strategic use of tactical deception (55), transitive inference (36), one-trial long-term memory (56), a strong ability to delay gratification (57), and MSR (18, 19).

Our study was conducted in the laboratory at the Department of Biology, Osaka City (Metropolitan) University, Japan. We used a total of 26 wild-captured cleaner fish purchased from a commercial ornamental aquarium dealer. Before arriving at the laboratory, these fish had been kept in isolation at the aquarium shop and were sent to the laboratory in separate containers. Fish were between 61 and 78 mm in total length (TL) with individuals of this size being functionally female (18). Each fish was housed individually in separate tanks (45 cm × 30 cm × 28 cm) and kept for at least 1 wk to be acclimated to captivity prior to the start of the experiments. Fish were kept in a 12:12-h light:dark cycle throughout the study. Each tank contained a PVC pipe as a sleeping shelter and a block of rock (10 cm × 5 cm × 5 cm) as a potential body-scraping site. Coral sand and coral pebble formed a 2- to 3-cm-thick substrate on the tank bottom. The water was aerated and filtered and temperature was kept between 25 and 26 °C. Fish were fed a small piece of diced fresh shrimp meat every day. These tank conditions were the same as in previous studies (18, 19).

### Description of Fish Behavior toward Mirror Reflection.

We attached a 45 × 28-cm^2^ high-quality mirror on one glass wall of the tank, which was initially completely obscured with a white plastic sheet (45 × 28 cm^2^). The methods of mirror presentation to subject fish were the same as those described in detail in Kohda et al. (18, 19). That is, at the start of the MSR test, the white sheet was removed from the mirror, exposing focal fish to their reflection until the end of the series of experiments (with the exception of several hours during control conditions when the mirror was temporarily covered; see below). All behaviors were video recorded (HDR-CX470, Sony, Tokyo, Japan) every day for 2h.

Kohda et al. (2019) (18) have described three stages of cleaner fish interaction with a mirror, characterized by these typical behaviors: i) socially aggressive behavior (mouth biting/fighting with the mirror image), ii) contingency or synchronicity testing behavior [atypical behaviors repeatedly performed in front of mirror; see supplementary videos in Kohda et al. 2019 (18)], and then iii) self-directed behavior (observing their reflection within 5cm from the mirror). The former two stages typically end after approximately 5 d of mirror exposure, and the observation of their reflection then begins (18). Since we observed the same sequence of behaviors as previous studies (18, 19), we used the start of this last stage as an indication that subjects were ready to pass the mark test. Thus, according to our criteria, we started to test subjects after 7 d of mirror exposure.

### Procedure of Mirror Mark Test.

We conducted the mirror mark tests on 10 cleaner fish (61 to 69 mm TL) after treatment 1 of experiment 1, as per the procedure of the original mark test by Kohda et al. (18, 19). On day eight of continuous mirror presentation, fish behavior was recorded for 2 h before any marking treatments. On the following night, we marked subjects with a brown-colored visual implant elastomer (VIE; Northwest Marine Technology Inc., Shaw Island, USA) subcutaneously on the throat. Fish behaviors were again recorded the following morning for 4 h beginning at 9 AM: the first 2 h while the mirror was covered (color marked without mirror) and the second 2 h with the mirror uncovered (color marked with mirror). The marking procedure did not alter fish behavior, and fish swam normally in the morning following the injection as they had done during previous studies (18, 19). All VIE marking, video recording, and video analyses for experiments 1 and 2 were conducted by N. Kubo and S. Sogawa. The analyses focused on the frequency of throat scraping on the rock or on the bottom substrate; events were counted by using video annotation software ELAN 5.2. Throat-scraping behaviors are not stereotypic actions in the cleaner fish but can vary between individuals (Movie S1 and refs. 18 and 19). Cleaner fish show the highest passing rate (94% of 17/18 fish) of the mirror mark test among species with large sample sizes. It has been speculated that the mark may resemble an ectoparasite and therefore acts as an “ecologically relevant mark,” which cleaner fish have a strong motivation to remove (19). Previous studies have shown that cleaner fish cannot feel the VIE color mark and do not sense the mark until they observe it visually (18, 19). Furthermore, other studies have quantified in detail swimming behavior in front of the mirror, proximity to the mirror, and general activity budgets (19), which we also summarize (Table 1).

We used cleaner fish that had passed the mirror mark test several months prior in experiment 2 (described below) but that were continuously exposed to a mirror in their home tank (45 cm × 28 cm × 30 cm). Fish were regularly video recorded to confirm that they were not acting aggressively toward their reflection during this time between experiments.

### Experiment 1: Self-Face Recognition.

In both treatments, 10 focal fish were kept individually in separate tanks (45 cm × 28 cm × 30 cm) for at least 1 wk in visual isolation to other fish. On day nine, all fish were anesthetized with 1/5,000 solution of anesthetic (FA100, Tanabe Pharmacy Inc.) and photographed outside of the tank using a digital camera (Nikon D610, Nikon, Tokyo, Japan).

Using these photograph images and software GIMP 2.2, we created four types of photographic models: self-face and self-body (SS model), unfamiliar face and unfamiliar body (UU model), self-face and unfamiliar body (SU model), and unfamiliar face and self-body (US model; Fig. 2). Since fish had been observing their mirror image, we reversed the self-face photograph to replicate the perspective that focal fish had been observing in the mirror. All photographs were size matched to each subject fish and printed on high-quality photo paper and laminated.

Photograph models were presented to focal fish on the outside of the aquarium glass, 15 cm above the tank bottom. A white plastic sheet (45 cm × 28 cm) had been placed outside the tank glass before the laboratory lights came on, and the photograph model was shown between the glass and the white sheet. Hence, focal fish could view but not touch the photograph models. Video cameras were set 60 cm from the front of the tank and photograph models were made visible around noon for 5 min with focal fish behavior video recorded.

#### Treatment 1: Before mirror exposure.

We examined whether mirror-naive fish could distinguish between a self-photograph and an unfamilar stranger’s photograph (Fig. 2*B*). SS models and UU models were randomly displayed to focal fish at 2 d intervals between trials. We predicted that both photographs would be attacked at similar frequencies by mirror-naive focal fish. One day after the last trial, a mirror was placed outside each tank to initiate the MSR test.

#### Treatment 2: After mirror exposure.

All subjects had been exposed to a mirror and passed the mirror mark test. The four types of photograph models were presented in a random order to focal fish with a 2 d interval period between experiments, i.e., on days 1, 4, 7, and 10. During the 10 d experimental period, the mirror was left uncovered, except for the period in the morning until model presentation at 12 noon. We predicted that mirror-experienced fish had by this stage developed a mental self-image and should now be able to distinguish between photographs (motionless images) of the self and of an unknown stranger (different strangers’ photographs were used before and after the mark test). In that case, they should not attack the self-photograph (SS model) but still frequently attack the photograph of an unknown fish (UU model) (Fig. 2). If cleaner fish have a mental image of the face, we also predicted that when viewing composite photograph models, they would not be aggressive to photographs with the self-face even if the photograph had an unknown stranger body (SU model), in contrast to photographs with the unfamilar face and the self-body (US model). After the first treatment and during the mark test, fish observed only self-image in a mirror, with no chance of establishing a mental image of another fish's face.

Aggressive behaviors directed toward photograph models were categorized into three types: i) rushing toward the photograph without touching glass, ii) physical bumping/attack by rushing toward the model and bumping the glass, and iii) biting. When biting, focal fish typically tried to quickly and repeatedly bite the glass/model photograph. Rushing behavior toward familiar and unfamiliar photographs was observed throughout the 5 min observation period.

### Experiment 2: Do Cleaner Fish Regard the Self-Face as a Familiar Individual?

In experiment 1, all cleaner fish that passed the mark test showed significantly less aggression toward the self-photograph compared with the unfamiliar photograph (Figs. 2 and 3). To test whether focal fish regard the self-photograph as the self or a very familiar individual, we designed an experiment exploiting the dear enemy relationship. Eight new clearer fish that had passed the mirror mark test were exposed to composite size-matched photographs of three (self, familiar, and unfamiliar) faces all on the body of another unknown individual. Photographs were shown to the focal fish for 5 min the next day of each treatment.

Previous studies have demonstrated that potential territorial rivals are tolerated by neighbors if they do not leave their territory, the “dear enemy relationship” (e.g., refs. 23, 31–33). However, if the rival betrays the relationship, e.g., leaving its territory for an extended period, the rival will be attacked when it returns by its neighbors. Kohda et al. (2022) (19) demonstrated that once cleaner fish have passed the mirror mark test, they no longer treat mirror images as a territorial rival and do not increase levels of aggression toward their self-image when it reappears at a different site (19). We exploited the dear enemy relationship to test whether cleaner fish can distinguish between photographs of the self and familiar individuals. We predicted that if cleaner fish can recognize the self-face, then aggression would not increase against self-photographs, in comparison with familiar photographs, after a period of absence from the focal fish’s neighboring territory. Being absent from the perceived territory makes focal fish consider neighbors to have betrayed the dear enemy relationship.

#### Treatment 1: Exposure to a familiar neighbor-face model.

We created familiar neighbors by placing pairs of tanks (45 cm × 30 cm × 28 cm) together to create four neighbor pairs. Initially, a white plastic sheet was placed between pairs of tanks for 7 d to allow fish to acclimate to their home tanks before viewing their neighbor. On day eight, the sheet was removed and interactions between pairs were video recorded for 1 h in the morning, noon, and in the evening and again at noon on days 9 and 10. Rates of aggression largely decreased with time (*SI Appendix*, Fig. S1); however, when pairs were swapped, aggression increased, demonstrating the dear enemy relationship had been established (*SI Appendix*, Fig. S1 and ref. 53).

The new pair combinations were kept for another 4 d, with aggression decreasing and pairs becoming tolerant of each other’s presence by the third day (*SI Appendix*, Fig. S1), demonstrating that dear enemy relationships had been established again. White sheets where then placed back between tanks so that neighbors were no longer visible. After 1 d (simulating betrayal of the dear enemy relationship), focal fish were exposed to a composite photograph model of their neighbor, and their behavioral reactions were video recorded for 5 min.

#### Treatment 2: Exposure to a self-face model.

We replicated the experimental procedure described above in treatment 1 but instead exposed focal fish to a mirror rather than a live neighbor. Mirrors were made visible, and focal fish behavior was video recorded for 2 h in the morning and again for 1 h around noon on days two and three. Focal fish were not aggressive toward their mirror reflection during the 3 d.

The mirror was covered on day four until the next morning, when focal fish were presented with a composite photograph of the self-face, and their behavioral responses were video recorded for 5 min. Under the dear enemy relationship, if cleaner fish regarded the self-face photograph instead as a familiar neighbor, then aggression should increase toward the self-face photograph. Half of the fish in this experiment first underwent treatment 1, while the remaining four first underwent treatment 2. Although the time exposed to a mirror was different between the two treatments, focal fish should regardless increase aggressiveness toward self-face photographs, if they perceived an increase in threat level, not due to familiarity.

#### Treatment 3: Exposure to a stranger-face model.

We also exposed cleaner fish to a composite photograph comprised of an unfamiliar face and unfamiliar body to examine aggressiveness toward an unknown stranger. We predicted that focal fish would act more aggressively toward an unfamiliar face photograph compared with a familiar face photograph.

We acknowledge that a sequence effect could have modulated focal fish aggressive behavior. However, we found no significant order effect in experiment 1 (negative binomial GLMM for attack frequency, full model: treatment x order: likelihood ratio test, χ^2^ = 10.64, df = 10, *P* = 0.39; reduced model without interactions, order: χ2 = 6.29, df = 4, *P* = 0.18; treatment: χ^2^ = 85.54, df = 4, *P* < 0.0001) and are therefore confident that we are observing biologically meaningful responses.

### Experiment 3: Photograph Mark Test.

When animals respond to a mark on their body that they viewed in a mirror reflection, they are regarded as having passed the mirror mark test. By using a mark placed on a photograph, we can also test whether animals recognize the self in a static image. We presented mark-naive cleaner fish (*n* = 8; 67.6 to 76.5 mm in size, mean = 71.2 mm) with self-photographs with a brown mark placed on the throat. These focal cleaner fish had been exposed to a mirror for 1 wk prior to the mark photograph experiment but had not undergone the mirror mark test. Focal fish were first presented with unmarked self-photographs for 5 min and then marked self-photographs for 1 h. As a control, on the following day, we presented the focal fish with an unmarked self-photograph for 5 min and then another unmarked self-photograph for 1 h. Experiments were purposely conducted in this order to prioritize the marked self-photograph treatment. We did not observe any difference in the frequency of body-scraping behavior compared with throat-scraping behavior between photographs with and without a mark (exact Wilcoxon signed-rank test, *V* = 13.5, *P* = 0.98, effect size = 0.066). We again reversed the self-face photographs prior to printing to replicate the perspective that focal fish had been observing in the mirror.

As an additional control, we also presented focal cleaner fish with a photograph model of a familiar fish that had a mark placed on the throat. We conducted this control to determine whether cleaner fish only react to a mark perceived to be on their own body or react with throat-scraping behavior to a mark observed on a familiar individual. We predicted that observing the marked model of the familiar individual would not elicit throat-scraping behavior in focal fish. To establish a familiar relationship between two fish, we placed two tanks adjacent to each other for 1 wk before (*n* = 4 fish) and after (*n* = 4) mirror presentation. First, we presented unmarked photographs of the familiar fish to focal fish for 5 min; we then presented marked photographs of familiar fish for 1 h, with the white plastic sheet used to obscure the view of focal fish between treatments. All focal fish behavior was video recorded.

### Behavioral Analyses.

In experiments 1, 2, and 3, focal fish aggression was video recorded for 5min and analyzed using video annotation software ELAN 5.2. Aggressive behaviors exhibited by cleaner fish were categorized into three categories: i) rushing toward perceived opponent with fins erected, ii) physical bumping/touching, and iii) biting. We quantified the number of these behaviors in each video recording. In mirror mark tests, we counted the number of throat-scraping behaviors performed in 2 h for each of E1: period before treatment, E2: fish marked but mirror not visible, and E3: fish marked and mirror visible (Fig. 1).

A subset of video recordings (5/16, each 60 min in length) were analyzed blind to reviewers (N. Kubo and T. Kobayashi) in experiment 3 to assess for any observer bias. The number of throat-scraping and body-scraping (including face) behaviors analyzed by both observers were highly correlated (throat: *r* = 0.999, *t* = 118.8, *P* < 0.0001; body: *r* = 1.00, *t* = 5.32, *P* <0.002, *n* = 5), suggesting no difference among observers. Furthermore, the timing (sec) of 38 body-scraping behaviors and one instance of throat scraping corresponded between observers (except in two cases of the former), indicating no observer bias influencing our results.

### Statistical Analyses.

All statistical analyses were performed using R version 4.1.1 (55). In experiment 1 (*n* = 10), we compared the frequencies of attacks by focal fish directed toward the various composite photographs before (SS and UU) and after fish passed the mirror mark test (model types: SS, SU, US, and UU). In experiment 2 (*n* = 8), we compared the frequencies of attacks by focal fish directed toward composite photographs of the self-face, a familiar face, and an unfamiliar face. In the mark test (*n* = 10), the number of throat-scraping behaviors was compared among the fish in E1: period of before treatment, E2: mark placed but mirror not visible, and E3: mark placed and mirror visible. In all the three experiments, we used Friedman tests and exact Wilcoxon signed-rank tests with the sequential Bonferroni correction procedure for post hoc comparisons to compare cleaner fish behavioral responses.

## Supplementary Material

Appendix 01 (PDF)

Dataset S01 (XLSX)

Dataset S02 (XLSX)

Dataset S03 (XLSX)

Dataset S04 (XLSX)

Dataset S05 (XLSX)

Dataset S06 (XLSX)

Dataset S07 (XLSX)

Movie S1. Throat scraping behaviors by a cleaner wrasse, *Labroides dimidiatus*, viewing a self-photograph with a color mark on the throat. This fish had passed the mirror mark-test two months prior and the color mark, which had been placed on the throat, had faded and was no longer visible. Moreover, this fish had not been observed exhibiting throat scraping behavior during this period. The mirror had been visible until the night before the video-recording. Outside of the tank (left side) a self-photograph was shown to the focal fish, with a mark placed on the throat. This focal fish scraped its throat 10 times during a 30 sec period. When viewing the self-photograph, this cleaner fish attempted to scrape its throat on a small block of sandy substrate three times. The fish viewed the photograph again and scraped the throat another four times. After again viewing the photograph, the fish scraped its throat three more times. Note that this fish tried to scrape the right side of its throat, coinciding with the mark being slightly on the left side of the throat in the photograph.

## Data Availability

All study data are included in the article and/or *SI Appendix*.
