# Supplementary material for: Cleaner fish recognize self in a mirror via self-face recognition like humans
Source: Proc Natl Acad Sci U S A. 2023 Feb 6;120(7):e2208420120. doi: 10.1073/pnas.2208420120 (PMC9963968; doi:10.1073/pnas.2208420120)
Supplement: Supplementary file 1 — Appendix 01 (PDF) [file pnas.2208420120.sapp.pdf]

Supplementary materials for

Cleaner fish recognize self in a mirror via self-face recognition like in humans

Masanori Kohda[1*], Redouan Bshary[2], Naoki Kubo[1], Satoshi Awata[1], Will Sowersby[1], Kento Kawasaka[1], Taiga Kobayashi[1], Shumpei Sogawa[1*]

Supplementary

Figure S1

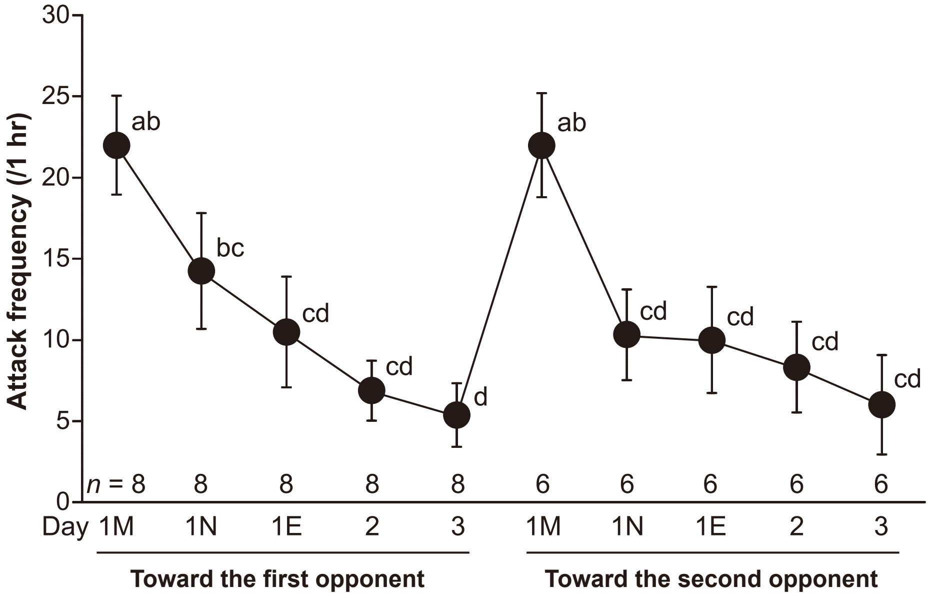

Caption of figure

Fig. S1. Results of the dear enemy experiment in cleaner fish. X axis: Morning (M), around noon (N), and evening (E) on the day 1, day 2, and day 3. Mean ± SEM. Due to the missing data of repeated measures (toward the second opponent), we used negative binomial generalized linear mixed models (GLMMs), with frequency of attacks as the response variable, time since the two fish first met as the independent variable, and focal fish ID as a random effect. Negative binomial GLMM, likelihood ratio test, $\chi^2 = 55.98$, df = 9, $P < 0.0001$. a, b, c, and d show statistical differences by Tukey's all-pair comparisons methods. These

analyses were conducted using *lmerTest*, *mass*, *multcomp*, and *lmtest* packages in R version 4.1.1.

Supplement movie

Supplement Movie S1: **Throat scraping behaviors by a cleaner wrasse *Labroides dimidiatus* viewing a self-photograph with a color mark on the throat.** This fish had passed the mirror mark-test two months prior and the color mark which had been placed on the throat had faded and was no longer visible. Moreover, this fish had not been observed exhibiting throat scraping behavior during this period. The mirror had been visible until the night before the video-recording. Outside of the tank (left side) a self-photograph was shown to the focal fish, with a mark placed on the throat.

This focal fish scraped its throat 10 times during a 30 sec period. When viewing the self-photograph, this cleaner fish attempted to scrape its throat on a small block and sandy substrate three times. The fish viewed the photograph again and scraped the throat another four times. After again viewing the photograph, the fish scraped its throat three more times. Note that this fish tried to scrape the right side of its throat, coinciding with the mark being slightly on the left side of the throat in the photograph.
